# Nanostructured ZnO as Multifunctional Carrier for a Green Antibacterial Drug Delivery System—A Feasibility Study

**DOI:** 10.3390/nano9030407

**Published:** 2019-03-11

**Authors:** Federica Leone, Roberta Cataldo, Sara S. Y. Mohamed, Luigi Manna, Mauro Banchero, Silvia Ronchetti, Narcisa Mandras, Vivian Tullio, Roberta Cavalli, Barbara Onida

**Affiliations:** 1 Politecnico di Torino, Department of Applied Science and Technology, Corso Duca Degli Abruzzi 24, 10129 Turin, Italy; federica.leone@polito.it (F.L.); roberta.cataldo@studenti.polito.it (R.C.); sara.mohamed@polito.it (S.S.Y.M.) luigi.manna@polito.it (L.M.); mauro.banchero@polito.it (M.B.); silvia.ronchetti@polito.it (S.R.); 2 Department of Public Health and Pediatrics, Microbiology Division, University of Turin, via Santena 9, 10126 Turin, Italy; narcisa.mandras@unito.it (N.M.); vivian.tullio@unito.it (V.T.); 3 Department of Drug Science and Technology, University of Turin, via Pietro Giuria 9, 10125 Turin, Italy; roberta.cavalli@unito.it

**Keywords:** Supercritical CO_2_, ibuprofen, NsZnO, antimicrobial activity

## Abstract

The physico–chemical and biological properties of nanostructured ZnO are combined with the non-toxic and eco-friendly features of the scCO_2_-mediated drug loading technique to develop a multifunctional antimicrobial drug delivery system for potential applications in wound healing. Two nanostructured ZnO (NsZnO) with different morphologies were prepared through wet organic-solvent-free processes and characterized by means of powder X-ray diffraction, field emission scanning electron microscopy (FESEM), and nitrogen adsorption analysis. The antimicrobial activity of the two samples against different microbial strains was investigated together with the in vitro Zn^2+^ release. The results indicated that the two ZnO nanostructures exhibited the following activity: *S. aureus* > *C. albicans > K. pneumoniae*. A correlation between the antimicrobial activity, the physico–chemical properties (specific surface area and crystal size) and the Zn^2+^ ion release was found. Ibuprofen was, for the first time, loaded on the NsZnO carriers with a supercritical CO_2_-mediated drug impregnation process and in vitro dissolution studies of the loaded drug were performed. A successful loading up to 14% *w*/*w* of ibuprofen in its amorphous form was obtained. A preliminary drug release test showed that up to 68% of the loaded ibuprofen could be delivered to a biological medium, confirming the feasibility of using NsZnO as a multifunctional antimicrobial drug carrier.

## 1. Introduction

Zinc oxide (ZnO) is a multifunctional material possessing unique physical and chemical properties, such as high chemical stability, a high electrochemical coupling coefficient, a broad range of radiation absorption and high photostability [1]. For these reasons it is largely used in many applications, ranging from electronics, optoelectronics, sensors and photocatalysis [2,3].

ZnO is also widely used in topical formulations to address several skin conditions, like burns, scars, and irritations, thanks to its non-toxicity, biocompatibility and antimicrobial activity [4].

ZnO exhibits three crystal structures named wurtzite, zinc-blend, and an occasionally noticed rock-salt [5], which allow it to be employed as a nanostructured material for different nanotechnology applications in many industrial areas, such as gas sensors, biosensors, semiconductors, piezoelectric devices, field emission displays and photocatalytic degradation of pollutants [6].

Due to the wide spread of nanotechnology, cosmetics and pharmaceuticals have also been revolutionized. Among all the materials, ZnO has been developed in different nanostructures to enhance its interaction with the skin and to improve the existing products. A promising application consists of the addition of ZnO in wound dressing materials. Nanocomposites represent a good example [7]: they consist of the addition of ZnO nanostructures to polymeric matrices [8,9,10,11] in order to impart novel functionalities, such as antibacterial activity. It is well-established in the literature that ZnO displays significant bactericidal properties over a broad range of bacteria [12,13]. This occurs due to several mechanisms, such as generation of reactive oxygen species (ROS), zinc ion release, membrane dysfunction, and nanoparticle penetration. Moreover the physico–chemical parameters of the nanomaterial, such as size, morphology, and specific surface area, remarkably affect the antibacterial properties of ZnO [14,15]. It has also been demonstrated that zinc ion release can improve wound healing [16,17], since zinc is an essential trace element in the human body and acts as a cofactor in zinc-dependent matrix metalloproteinases that augment auto debridement and keratinocyte migration during wound repair.

The treatment of painful wounds is another important issue in biomedicine. It has been demonstrated that painful wounds can take more time to heal, leading to a lack of compliance by the patients. Several research works [18] have addressed the development of innovative wound dressings, able to deliver small doses of anti-inflammatory analgesic drugs to the wound [19]. In this context the use of ZnO as a drug carrier to be included in the wound dressing device could be of particular interest thanks to its outstanding biocompatibility, even though the application of this material as a drug delivery system has not been investigated widely in the literature [20] and may be considered at its nascent stage.

The use of organic solvents in pharmaceutical technologies is another challenging issue since it leads both to health concerns, which are related to the toxicity of residual solvents in the final products, and to a negative environmental impact. In the last decade, supercritical fluid technology has been emerging as a green drug impregnation method [21]. Supercritical carbon dioxide (scCO_2_) is the most used supercritical solvent because it is readily available, cheap, non-toxic, non-flammable, and recyclable. At the end of the scCO_2_–mediated drug impregnation process a simple depressurization step allows a ready-to-use organic-solvent-free drug loaded material to be obtained. Furthermore, it offers the possibility to tailor the operating parameters of the impregnation process, such as temperature, pressure, and time, on the basis of the selected drug/carrier system [22]. This permits a better drug/carrier interaction to be obtained, with the drug in an amorphous state, which improves its dissolution profile and, consequently, its bioavailability [23].

Notwithstanding the above reported remarkable advantages, some drawbacks in the use of this technology have emerged, such as the scarce ability of scCO_2_ to dissolve polar and ionic species, since it is a linear molecule with no net dipole moment. Furthermore, the elevated pressure required and high maintenance cost can represent a limitation in the use of this technology [21].

Even though the incorporation of active pharmaceutical ingredients (APIs) in organic and inorganic carriers through scCO_2_ has been proposed in different research areas [22,24,25], the loading of drugs on ZnO carriers has not been investigated yet [23] and, to the best of our knowledge, the clotrimazole incorporation described in our previous work [26] represents the very first study about the loading of an API on nanostructured ZnO by means of scCO_2_.

The fundamental idea of this research project is to combine the physico–chemical and biological properties of ZnO with the eco-friendly features of the scCO_2_–mediated drug impregnation process to develop a green multifunctional device for treating painful wounds. This consists of the combination of antibacterial and anti-inflammatory/analgesic action in a single delivery system. ZnO is particularly suitable for this role, because its nanostructure can be tailored to host drug molecules and because it can offer intrinsic antimicrobial activity [15]. Ibuprofen (IBU) has been selected as the drug to be hosted in the ZnO nanostructures, since it is one of the most commonly used and most frequently prescribed non-steroidal anti-inflammatory drug (NSAID) for oral and topical administration due to its prominent analgesic role [27,28], and it has already been employed to prepare innovative pain-reducing wound dressings [18,19]. Furthermore IBU has also been widely used in many scCO_2_–mediated drug impregnation processes [23].

This work is a feasibility study aiming at investigating the possibility of loading IBU on different ZnO carriers by means of scCO_2_ and checking the antimicrobial activity as well as the capability of the obtained system to release Zn^2+^ ions and the drug, which are essential requirements for the development of a multifunctional device for wound healing applications. Two nanostructured ZnO (NsZnO) powders with different morphologies and physico–chemical parameters were synthetized through wet organic-solvent-free processes [26] and characterized by means of powder X-ray diffraction, FESEM images, and nitrogen adsorption analysis. The samples were also characterized from a biological point of view; particularly, their antimicrobial activity against different microbial strains and the in vitro Zn^2+^ release profiles from the NsZnO matrices were evaluated. IBU was loaded on the NsZnO carriers with a scCO_2_–mediated drug impregnation process and in vitro dissolution studies of the loaded drug were performed.

## 2. Materials and Methods

### 2.1. Materials

All the reagents for chemical synthesis, as well as ibuprofen, were purchased from Sigma-Aldrich and used as received without further purification. Carbon dioxide with a purity of 99.998% was supplied by SIAD (Italy). Bidistilled water was used throughout this study.

### 2.2. Synthesis of Nanostructured ZnO (NsZnO)

Two different NsZnO materials were synthesized as previously described [26], using two different organic solvent free processes: the first one was based on the use of sole water as the solvent, i.e., a chemical bath deposition (NsZnO-1) method, while the second was a soft-template sol–gel synthesis method (NsZnO-2).

### 2.3. Drug Adsorption from Supercritical Carbon Dioxide

**Figure 1:**
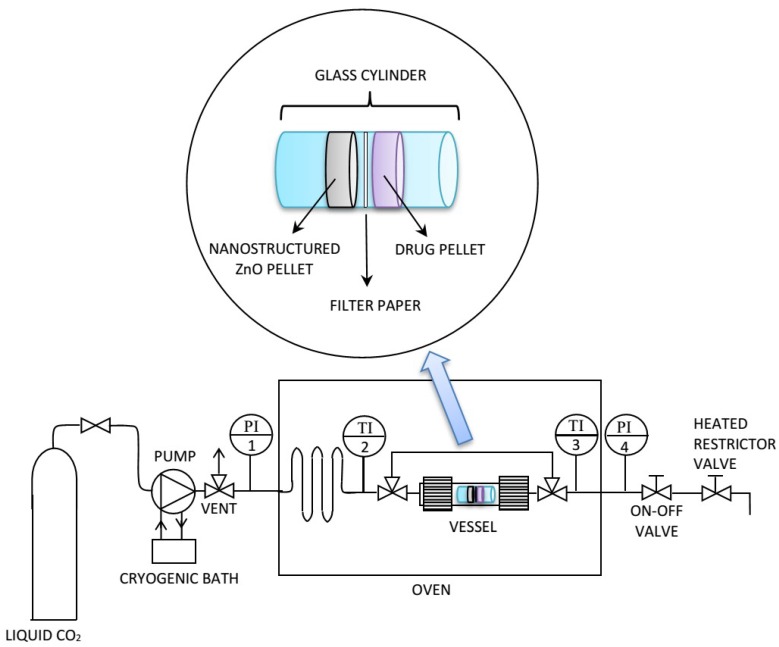
Experimental apparatus for supercritical carbon dioxide (scCO_2_)-mediated drug loading.

The scCO_2_–mediated drug loading was carried out using a procedure that had been developed in previous works [26,29]. It consisted of contacting the drug and each of the two NsZnO materials in a static atmosphere of scCO_2_ at constant temperature and pressure (Figure 1). First, a pellet of the drug (100 mg) and a pellet of the NsZnO (100 mg) were prepared and introduced into a glass cylinder of 1 cm diameter. A disc of filter paper was placed between the two pellets to prevent their contact and guarantee an efficient recovery of the samples at the end of the drug loading process. The glass cylinder was placed inside a stainless-steel vessel, which was put in an oven that kept the entire system at constant temperature. Liquid CO_2_ was used to fill the vessel, then the temperature was increased to 35 °C and additional CO_2_ was pumped to reach the target pressure (10 MPa). The pump was coupled with a cryogenic bath to prevent cavitation. The system was maintained at the above-reported conditions for 12 h. At the end of the drug loading process, the on–off valve was opened, and the apparatus was brought back to atmospheric pressure by means of a heated restrictor valve.

The IBU-containing materials are denoted hereafter as IBU@NsZnO-1 and IBU@NsZnO-2.

Moreover, the two carriers as such were treated in the same conditions in the absence of IBU in the glass cylinder, in order to investigate the effect of the scCO_2_ treatment on the NsZnO samples.

### 2.4. Morphological Characterization

FESEM images were recorded with an FESEM Zeiss Merlin instrument, equipped with an EDS detector (Oxford Instruments, Abingdon-on-Thames, UK).

### 2.5. Powder X-ray Diffractometry

XRD patterns were obtained using a Panalytical X’Pert (Cu Kα radiation, Almelo, The Netherlands) diffractometer. Data were collected with a 2D solid state detector (PIXcel) from 20 to 70 2θ with a step size of 0.001 2θ and a wavelength of 1.54187 Å.

### 2.6. Nitrogen Adsorption Analysis

Nitrogen adsorption isotherms were measured using a Quantachrome AUTOSORB-1 instrument (Boynton Beach, FL, USA). Before the adsorption measurements, samples were outgassed for 2 h at 100 °C. BET specific surface areas (SSA_BET_) were calculated in the relative pressure range of 0.04–0.1.

### 2.7. Thermogravimetry Analysis

Thermogravimetry (TG) analyses were carried out between 20 °C and 800 °C in air (flow rate 100 mL/min with a heating rate of 10 °C/min) using a SETARAM 92 instrument (Caluire et Cuire, France). 

### 2.8. Antimicrobial Activity of NsZnO

#### 2.8.1. Microbial Strains and Culture Conditions

The antibacterial activity of NsZnO-1 and NsZnO-2 was tested against a Gram-positive and a Gram-negative bacterial strain, such as *Staphylococcus aureus* ATCC 29213 and *Klebsiella pneumoniae* ATCC 700603, respectively. The antifungal activity of NsZnO-1 and NsZnO-2 samples was investigated against *Candida albicans* ATCC 90023. The strains were purchased from American Type Culture Collection (ATCC) (Manassas, VA, USA).

#### 2.8.2. Inocula Preparation

Microorganism inocula were prepared by picking two to three colonies from an overnight culture of *S. aureus/K. pneumoniae* on Brain heart infusion agar (BHA, Merck KGaA, Darmstadt, Germany) or of *C. albicans* on Sabouraud dextrose (SAB, Merck KGaA, Darmstadt, Germany) agar at 37 °C (bacteria) or 35 °C (yeasts), suspending them in 5 mL of 0.85% normal saline, to yield a 0.5 McFarland turbidity standard, corresponding to a suspension of ~5 × 10^8^ CFU/mL for bacteria or 5 × 10^6^ CFU/mL for yeasts.

Bacterial suspensions were diluted 1:1000 in Mueller Hinton broth (MHB, Merck KGaA, Darmstadt, Germany) to obtain a final concentration of 10^5^ CFU/mL. Fungal suspension was diluted 1:1000 in RPMI-1640 without sodium bicarbonate and with L-glutamine (Invitrogen, San Giuliano Milanese, Milano, Italy), buffered to pH 7.0 with 0.165 M morpholinepropanesulfonic acid (MOPS) (Sigma-Aldrich, Milan, Italy), and supplemented with glucose 0.2%, to obtain a concentration of 10^3^ CFU/mL. Inocula were confirmed by colony counts in duplicate.

#### 2.8.3. In Vitro Antimicrobial Assays

Determination of Minimum inhibitory concentration (MIC), Minimum bactericidal concentration (MBC), and Minimum fungicidal concentration (MFC).

The antimicrobial activity of NsZnO-1 and NsZnO-2 was determined using a broth microdilution (BM) method susceptibility assay, according to Clinical and Laboratory Standard Institute guidelines (CLSI document M07-A9 for bacteria, and M27-A3 for yeasts) [30,31]. As guidelines were not available for susceptibility testing of NsZnO, the antimicrobial activity was assessed following the CLSI BM method, with some modifications.

MIC determination was performed by serial dilution using 96-well microtiter plates (Sarstedt, Milan, Italy). Stock suspensions of NsZnO prepared at 30,000 µg/mL (*w*/*v*) in phosphate buffered solution (PBS; pH 7.4) were dispersed for 1 h using an ultrasonic bath, in order to minimize sedimentation of NsZnO particles. Doubling dilutions of the ZnO ranging from 15,000 to 30 µg/mL were prepared in 96-well microtiter plates in MHB for bacteria or in RPMI-1640 with MOPS for yeasts. After inoculum addition (0.1 mL), the plates were incubated under normal atmospheric conditions at 37 °C (bacteria) or 35 °C (yeasts) for 24 h. A sterile medium incubated under the same conditions was used as a blank, while the medium inoculated with the target microorganisms (without NsZnO) was used as a positive control of growth. All determinations were performed in duplicate. The lowest concentration of the NsZnO showing complete inhibition of visible growth was defined as MIC.

MBC and MFC of NsZnO were determined by subculturing 10 μL of broth taken from all the wells without visible growth onto BHA (bacteria) or SAB (yeasts) agar plates that did not contain the test agents. After incubation for 24 h at 37 °C (bacteria) or 35 °C (yeasts), MBC or MFC were defined as the lowest concentration of ZnO resulting in the death of 99.9% of the inoculum in no subculture [32].

##### Viable microorganism counts

To assess the antimicrobial activity of NsZO over time, the number of viable microorganisms was measured by monitoring bacterial/fungal growth after 24 h [33].

Briefly, the bacterial or yeast cells were grown overnight in BHI (Merck KGaA) or Sabouraud Dextrose (SAB, Merck KGaA, Darmstadt, Germany) broth at 37 °C or 35 °C, respectively. Bacteria and/or yeasts were harvested by centrifugation, washed, suspended in PBS, and diluted to yield a stock suspension of ~5 × 10^5^ CFU/mL. All the NsZnO samples with concentration of 15,000 µg/mL, suspended in PBS, were incubated with bacterial or yeast suspension in a shaker incubator at 37 °C or 35 °C, respectively, for 24 h. PBS solution was used as a negative control. All samples were serially diluted and 100 µL of bacterial/yeasts suspension was drawn from each sample tube, spread on BHA or SAB agar plates, and incubated at 37 °C or 35 °C for 24 h, so that the number of CFU/mL could be determined.

### 2.9. In Vitro Zinc Ions Release

Zinc ion release from the samples NsZnO-1 and NsZnO-2 was studied using vertical Franz diffusion cells and synthetic skin (Dow Corning, 7-4107, Silicone Elastomer Membrane, Biesterfeld Polychem, Milan, Italy). Suspensions of NsZnO (5 mg of powder in 0.5 mL of PBS buffer solution) were employed as the donor phases. The receiving phase consisted of a PBS buffer solution at pH 7.4. The apparatus was maintained under stirring at 33 °C, during which, at scheduled time intervals, the receiving phase was withdrawn and entirely substituted with a fresh receiving phase. Zinc ion quantification was performed in each withdrawn sample using inductively coupled plasma mass spectrometry (ICP-MS, Thermo Scientific, Waltham, MA, USA).

### 2.10. Preliminary In Vitro Drug Release Study

The ability of the IBU-loaded NsZnO to release IBU was tested by using a multicompartment rotating cell equipped with a hydrophilic dialysis membrane (Spectra/Por, Spectrum^®^, cut-off 12,000–1,4000 Da, Sigma-Aldrich, Milan, Italy). PBS solution (1 mL) was used as the receiving medium. At predetermined time intervals, the receiving phase was completely replaced by a fresh solution, and analyzed for IBU content at 263 nm, using a Beckman–Coulter DU 730 Spectrophotometer (Indianapolis, IN, USA).

## 3. Results and Discussion

**Figure 2:**
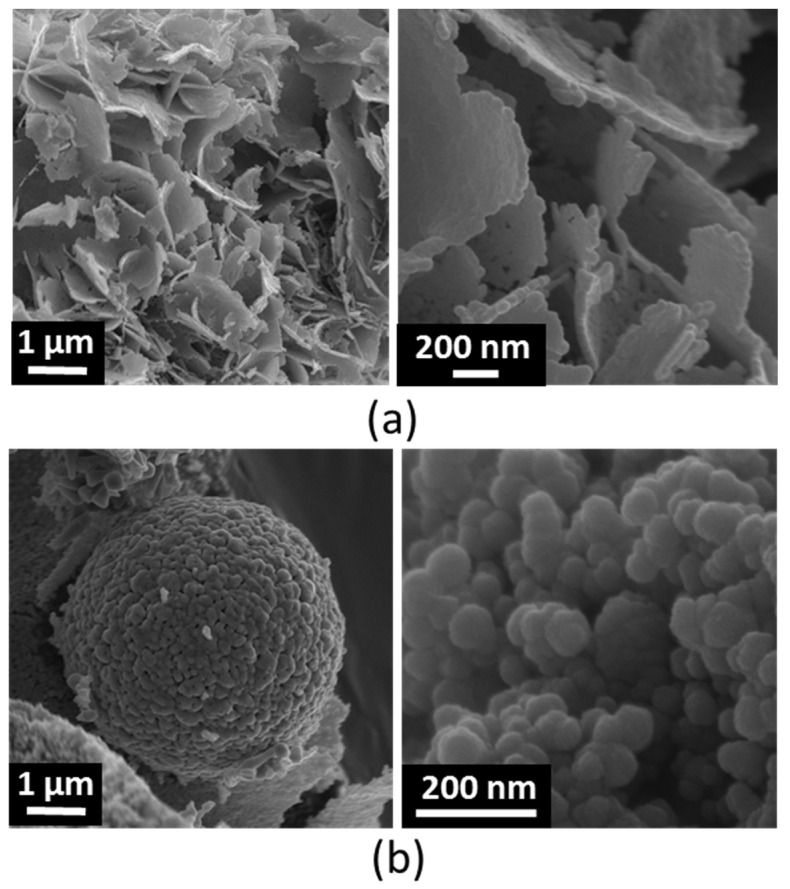
FESEM images of nanostructured (Ns)ZnO-1 (**a**) and NsZnO-2 (**b**).

Figure 2 shows the FESEM pictures of NsZnO-1 and NsZnO-2. As observed in the previous study [26], the two carriers were characterized by different morphologies.

NsZnO-1 appeared in the form of aggregates of nanosheets, with a thickness of about 20 nm, that were formed by the self-assembling of ovoid nanoparticles (having sizes around 15–20 nm). This morphology is in agreement with the mechanism growth proposed by Kakiuchi et al. [34].

The morphology of NsZnO-2 consisted of micrometric and sub-micrometric aggregates of nanoparticles with heterogeneous sizes of tens of nanometers.

Primary nanoparticles of NsZnO-1 were definitely smaller than those of NsZnO-2. The values of BET specific surface area and pore volume obtained by nitrogen adsorption are shown in Table 1. As previously observed [26], both features were larger for NsZnO-1 (68 m^2^/g and 0.230 cm^3^/g, respectively) than for NsZnO-2 (12 m^2^/g and 0.050 cm^3^/g, respectively), due to the lower particles size of NsZnO-1.

**Figure 3:**
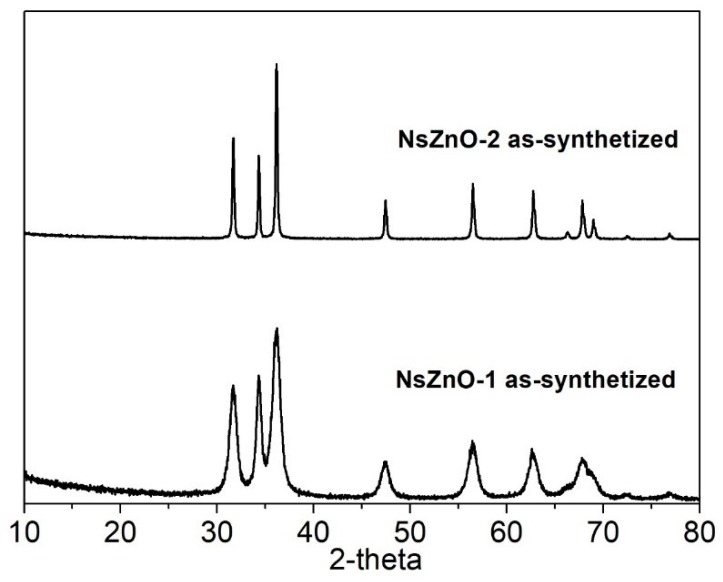
XRD patterns of NsZnO-1 and NsZnO-2.

Figure 3 reports the XRD patterns of both NsZnO materials, which reveal the occurrence of a highly crystalline single hexagonal phase of a wurtzite structure (JCPDS ICDD 36-1451). Comparing the XRD patterns of the two samples, it is evident that NsZnO-1 showed broader peaks than NsZnO-2, in agreement with the smaller particles size evidenced by FESEM analyses.

**Figure 4:**
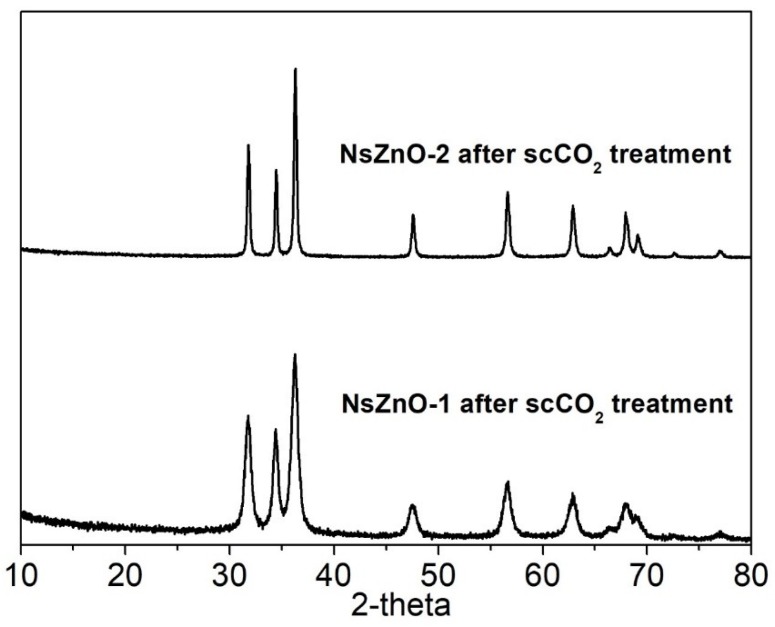
XRD patterns of NsZnO-1 and NsZnO-2 after scCO_2_ treatment.

In order to investigate the stability of NsZnO carriers in scCO_2_, the XRD patterns of NsZnO-1 and NsZnO-2 after treatment in scCO_2_ for 12 h, at 35 °C and 10 MPa, were collected and these are reported in Figure 4. In both cases the hexagonal wurtzite pattern of ZnO was preserved, and no new peaks were detected. This result showed that no extensive reaction between ZnO and the CO_2_ occurred, which should not have been taken for granted, considering that the reaction between ZnO and CO_2_ to give ZnCO_3_ is a well-known phenomenon [35,36]. This evidence confirms the feasibility of using scCO_2_ as a solvent for the drug loading of NsZnO carriers.

As far as the IBU loading is concerned, its content was calculated as the weight loss by TG analysis (Figure S1) and was found equal to 14% *w*/*w* for IBU@NsZnO-1 and 9% *w*/*w* for IBU@NsZnO-2, respectively (Table 2).

The larger IBU content in IBU@NsZnO-1 than in IBU@NsZnO-2 was ascribed to the larger specific surface area and pore volume of NsZnO-1, which yielded larger drug adsorption and loading capacity.

Due to the presence of IBU molecules on the NsZnO carriers, the specific surface area and pore volume drastically decreased in both systems, as revealed by the data reported in Table 1.

It is worth noting that IBU contents in the two systems were similar to those previously obtained for clotrimazole adsorbed by scCO_2_ on NsZnO-1 and NsZnO-2 carriers [26], which were equal to 17% *w*/*w* and 14% *w*/*w*, respectively. This suggests the robustness of the scCO_2_ approach in the drug loading of NsZnOs.

**Figure 5:**
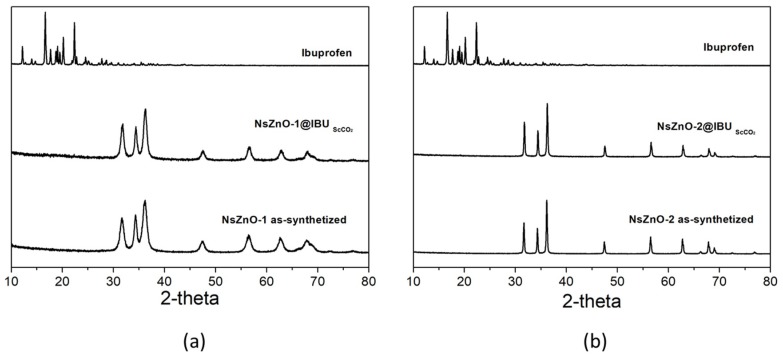
(**a**) XRD patterns of NsZnO-1, IBU@NsZnO-1, and pure crystalline IBU. (**b**) XRD patterns of NsZnO-2, IBU@NsZnO-2, and pure crystalline IBU.

XRD analyses were carried out to characterize IBU@NsZnO-1 and IBU@NsZnO-2. Figure 5 reports the XRD patterns of both systems, in comparison with those of the materials as-such and the pure crystalline IBU. No additional diffraction peaks typical of the crystalline IBU were observed for either IBU@NsZnO-1 or IBU@NsZnO-2 samples. This reveals that drug molecules are not assembled in the crystalline form on the two carriers.

The same result was previously obtained in the case of clotrimazole [26]. The amorphous form of the drug adsorbed on NsZnO from scCO_2_ may be ascribed to the scCO_2_–mediated process, which is known to favor the amorphization of the adsorbed drug [23]. This is a crucial aspect, in particular for poorly water-soluble drugs, because it is widely accepted that amorphization of the drug molecules plays a key role in increasing their dissolution rate and solubility.

**Figure 6:**
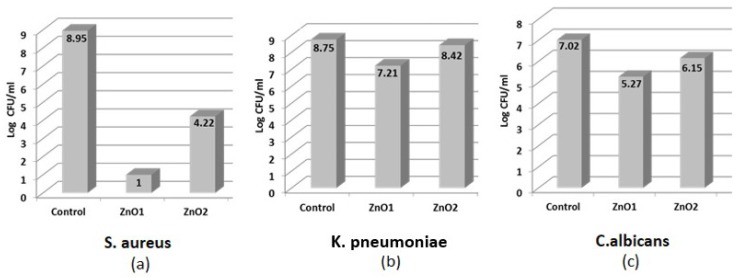
Comparison of antibacterial activities of NsZnO-1 and NsZnO-2 against *S. aureus* (**a**), *K. pneumoniae* (**b**), and *C. albicans* (**c**) expressed in Log CFU/mL.

In order to investigate the antimicrobial activity of NsZnOs, some microbiological parameters, such as MIC, MBC, and MFC were used (Table 3). In addition, a CFU assay was used to measure the antimicrobial activity of the NsZnOs over time by monitoring bacterial/fungal growth within 24 h (Table 4, and Figure 6).

As emerged from data shown in Table 3 the two ZnO nanostructures exhibited a stronger activity on the Gram-positive *S. aureus* than the Gram-negative *K. pneumoniae*. Between the two ZnO nanostructures, NsZnO-1 showed better activity than NsZnO-2 against *S. aureus* with an MIC value of 120 µg/mL vs 230 µg/mL. Since ZnO suspensions appeared “cloudy” in the case of *C. albicans*, it was not possible to determine the MIC from the visual appearance of the medium; hence, MFC was not assessed for this yeast (data not shown). In general, MBCs were two concentrations higher than MICs, with exception of NsZnO-2 that showed a MBC value one concentration higher than MIC against *S. aureus*, suggesting a more bacteriostatic activity of these compounds.

Table 4 and Figure 6 report the results of the viable microorganism counts assessed through a CFU assay. The bactericidal activity of NsZnO-1 against *S. aureus* (expressed in Log CFU/mL) was greater than that achieved by NsZnO-2 (1 vs 4.22, Figure 6a). The same trend, even if with less microbial load reduction values, is evident in Figure 6b for *K. pneumoniae*, where the Log CFU/mL of bacterial load was 7.21 and 8.42 for NsZnO-1 and NsZnO-2, respectively. Despite the failure of the broth dilution technique for yeasts, the enumeration of viable organisms’ method was efficient in the determination of the antifungal activity. The results are shown in Figure 6c. NsZnO-1 log counts observed for *C. albicans* was 5.27, whereas NsZnO-2 was able to reduce the yeast cells growth of 6.15 log in comparison with ZnO-free controls (7.02 log). Taken together, these results indicate that the two ZnO nanostructures exhibited a better activity towards *S. aureus* than *K. pneumoniae* and *C. albicans*.

Our data are difficult to compare due to the different methods and microorganisms used in the antimicrobial activity determination. However, these data are in agreement with those of some authors who detected a better antimicrobial activity of ZnO-compounds on Gram positive than Gram negative bacteria, and a good antifungal activity on *C. albicans* [13,37,38].

In addition, our results agree with the conclusions of Reddy et al. [39] and Tayel et al. [40] and disagree with the conclusions of Pasquet et al. [14]. In detail, Reddy and Tayel explained that the peptidoglycan cell-wall of Gram-positive bacteria may promote ZnO attachment onto the cell wall, whereas cell-wall lipophilic components of Gram-negative bacteria may oppose this attachment. Until now, the antifungal activity mechanism has not been well clarified; however, the candidacidal mechanism of ZnO can be probably ascribed to the cellular structure disruption or to inhibition of biological molecular synthesis due to Zn^2+^ release [38].

Among the NsZnOs investigated, the sample NsZnO-1 showed higher antimicrobial activity compared to NsZnO-2. This trend was confirmed by both the in vitro tests. This phenomenon could be ascribed to the crystallite sizes of the nanoparticles, which have been reported to greatly impact their antimicrobial activity, probably because of a greater accumulation of the nanoparticles inside the cell membrane and cytoplasm [12]. In fact, NsZnO-1 is characterized by lower crystallite sizes than NsZnO-2. This observation can be reinforced by the results obtained with *C. albicans,* against which NsZnO-1 showed better antifungal activity than NsZnO-2. These conclusions are consistent with the study of Lipovsky et al. [41], who suggested that ZnO nanoparticles display a marked activity against *C. albicans* and that the cytotoxic effect is size dependent.

Among the key mechanisms influencing the antimicrobial activity of nanostructured ZnO, it is important to consider the release of Zn^2+^ ions.

**Figure 7:**
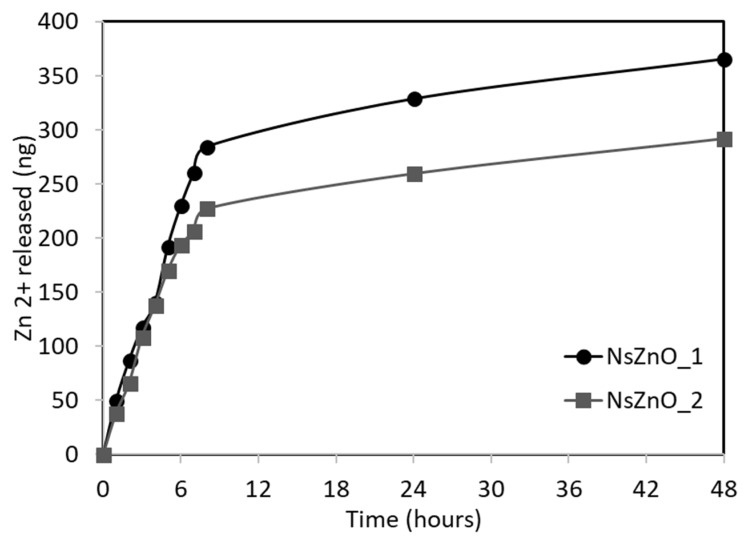
In vitro Zn^2+^ release from NsZnO-1 and NsZnO-2.

For this reason, a simple test was carried out to study the zinc ion release from NsZnO-1 and NsZnO-2 using vertical Franz diffusion cells equipped with synthetic skin. The results drawn from the zinc ion quantification are shown in Figure 7. The in vitro Zn^2+^ release study evidenced the ability of both the NsZnOs to release Zn^2+^, highlighting their potential use as multifunctional antimicrobial drug carriers.

A higher amount of Zn^2+^ ion was released from NsZnO-1 and this was ascribed to the lower crystallite size and the higher SSA of the sample. The maximum amount of released Zn^2+^ ion after 48 h corresponded to a percentage by mass of zinc equal to about 0.009% for NsZnO-1 and 0.007% for NsZnO-2: these low values confirm that the release is a surface phenomenon.

The higher Zn^2+^ ion release ability of NsZnO-1 is in agreement with the higher antibacterial activity of this carrier (Figure 6).

The in vitro ibuprofen release study was aimed at verifying the possibility of releasing the drug from IBU@NsZnO-1 and IBU@NsZnO-2 systems, assessing the lack of complete irreversible trapping of drug molecules in the carrier. The same test was carried out with crystalline ibuprofen for comparison.

**Figure 8:**
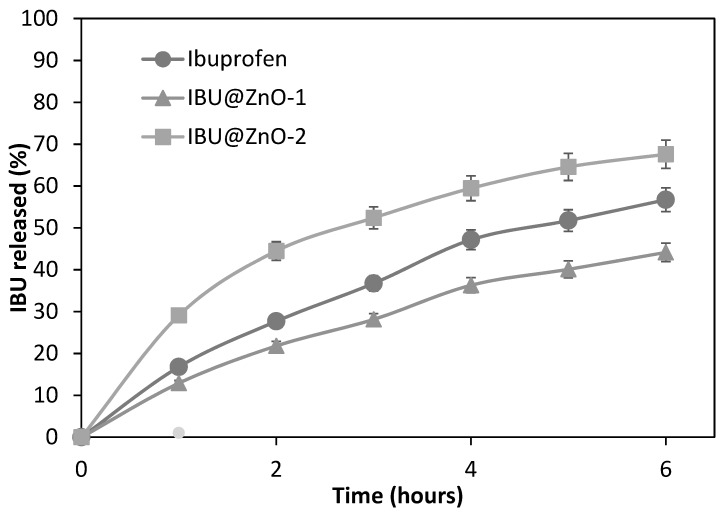
In vitro release profile of ibuprofen from IBU@NsZnO-1, IBU@NsZNO-2, and crystalline ibuprofen.

Figure 8 shows the cumulative release curves of ibuprofen. The percentage of drug released in 6 h was 68% for IBU@NsZnO-2, 44% for IBU@NsZnO-1, and 57% for crystalline ibuprofen.

These data reveal that both NsZnO-1 and NsZnO-2 were able to act as carriers for ibuprofen delivery. The different percentage of IBU released from the two materials may be ascribed to the different morphology and pore volume, which affect the distribution of drug molecules in the carrier and their diffusion to the receiving solution.

In conclusion, this preliminary in vitro release test showed that ibuprofen adsorbed on the NsZnO-1 and NsZnO-2 by scCO_2_ can be delivered, confirming the potential role of these nanostructures as drug delivery systems, as previously observed in the case of clotrimazole [26].

## 4. Conclusions

A feasibility study was conducted to investigate the possibility of developing a green multifunctional device for wound healing applications. Two multifunctional drug delivery systems based on nanostructured ZnO were prepared by means of non-toxic and organic-solvent free procedures.

The antimicrobial properties of the ZnO carriers were investigated against both Gram-positive and Gram-negative bacterial strains, such as *S. aureus* and *K. pneumoniae*, as well as against *C. albicans*. As a whole, the results indicated that the two ZnO nanostructures exhibited the following activity: *S. aureus* > *C. albicans > K. pneumoniae*.

Moreover, an in vitro Zn^2+^ release study was carried out. A correlation between the antimicrobial activity, the physico–chemical properties (specific surface area and crystal size) of nanostructured ZnO and the Zn^2+^ ion release was found.

For the first time ibuprofen was successfully loaded on the nanostructured ZnO carriers with a supercritical CO_2_-mediated drug impregnation process. The drug-loaded amount was observed to depend on the specific surface area and the pore volume of the carrier and up to 14% *w*/*w* of ibuprofen in its amorphous form was obtained inside the final drug delivery system. A preliminary drug release test showed that up to 68% of the loaded ibuprofen could be delivered to a biological medium, confirming the feasibility of using nanostructured ZnO as a multifunctional antimicrobial drug carrier.

## Figures and Tables

**Table 1:** Specific surface area (SSA) and pore volume values before and after the ibuprofen (IBU) adsorption by scCO_2_ process.

	Before IBU Adsorption		After IBU Adsorption	
	SSA_BET_ (m^2^/g)	Pore Volume (cm^3^/g)	SSA_BET_ (m^2^/g)	Pore Volume (cm^3^/g)
**NsZnO-1**	68	0.230	8	0.04
**NsZnO-2**	12	0.050	nil	nil

**Table 2:** Ibuprofen content in IBU@NsZnO-1 and IBU@NsZnO-2.

	IBU Content (% *w*/*w*)
IBU@NsZnO-1	14
IBU@NsZnO-2	9

**Table 3:** Minimum inhibitory concentration (MIC), and minimum bactericidal concentration (MBC), of NsZnO-1 and NsZnO-2 determined for *S. aureus*, *K. pneumoniae*, and *C. albicans* expressed in µg/mL. Minimum fungicidal concentration (MFC) for *C. albicans* was not determined.

Microbial Strain	*S. aureus*		*K. pneumoniae*		*C. albicans*
	MIC (µg/mL)	MBC (µg/mL)	MIC (µg/mL)	MBC (µg/mL)	MIC (µg/mL)
NsZnO-1	120	>470	470	1875	>15,000
NsZnO-2	230	>470	930	>3750	>15,000

**Table 4:** Comparison of antibacterial/antifungal activity of NsZnO-1 and NsZnO-2 against *S. aureus*, *K. pneumoniae*, and *C. albicans* determined with the enumeration of viable microorganism assay and expressed in CFU/mL after 24 h of incubation.

Microbial Strain	*S. aureus*	*K. pneumoniae*	*C. albicans*
NsZnO-1	10 CFU/mL	1.62 × 10^7^ CFU/mL	1.85 × 10^5^ CFU/mL
NsZnO-2	1.65 × 10^4^ CFU/mL	2.66 × 10^8^ CFU/mL	1.43 × 10^6^ CFU/mL

## Supplementary Materials

The following are available online at https://www.mdpi.com/2079-4991/9/3/407/s1, Figure S1: Section A: TG curves of NsZnO-1 and IBU@NsZnO-1; Section B: TG curves of NsZnO-2 and IBU@NsZnO-2.
