# Supplementary figures and images for: Nanostructured ZnO as Multifunctional Carrier for a Green Antibacterial Drug Delivery System—A Feasibility Study
Source: Nanomaterials (Basel). 2019 Mar 11;9(3):407. doi: 10.3390/nano9030407 (PMC6473990; doi:10.3390/nano9030407)

(A)

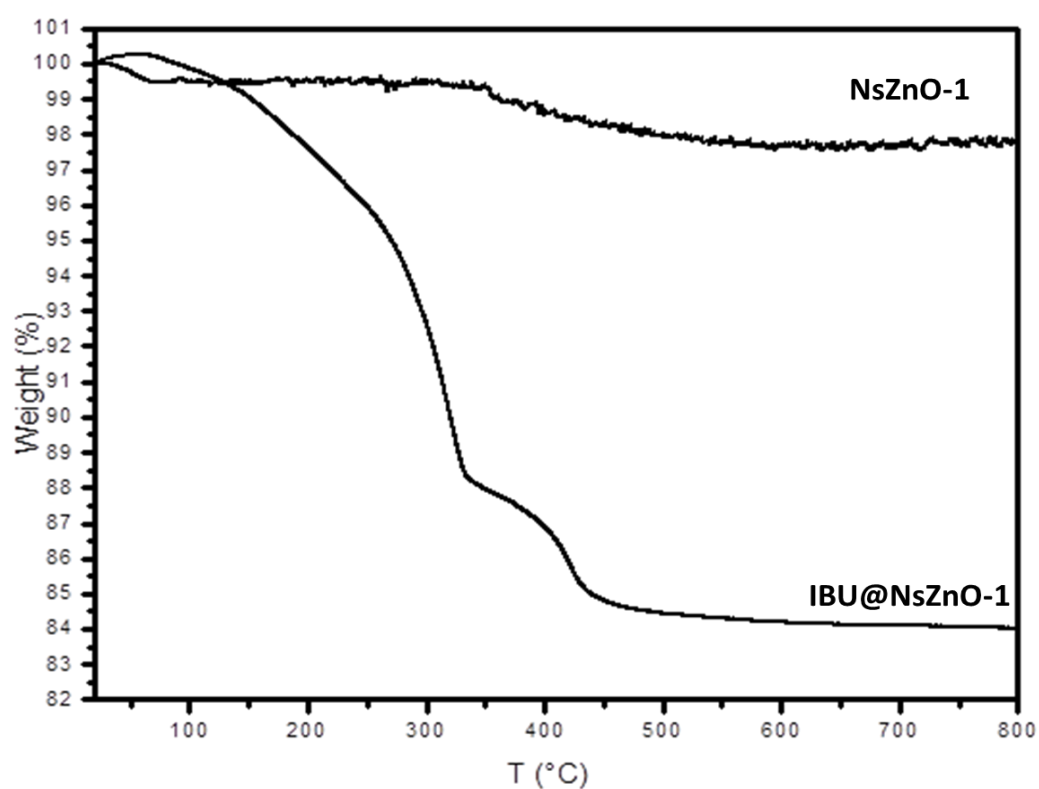

(B)

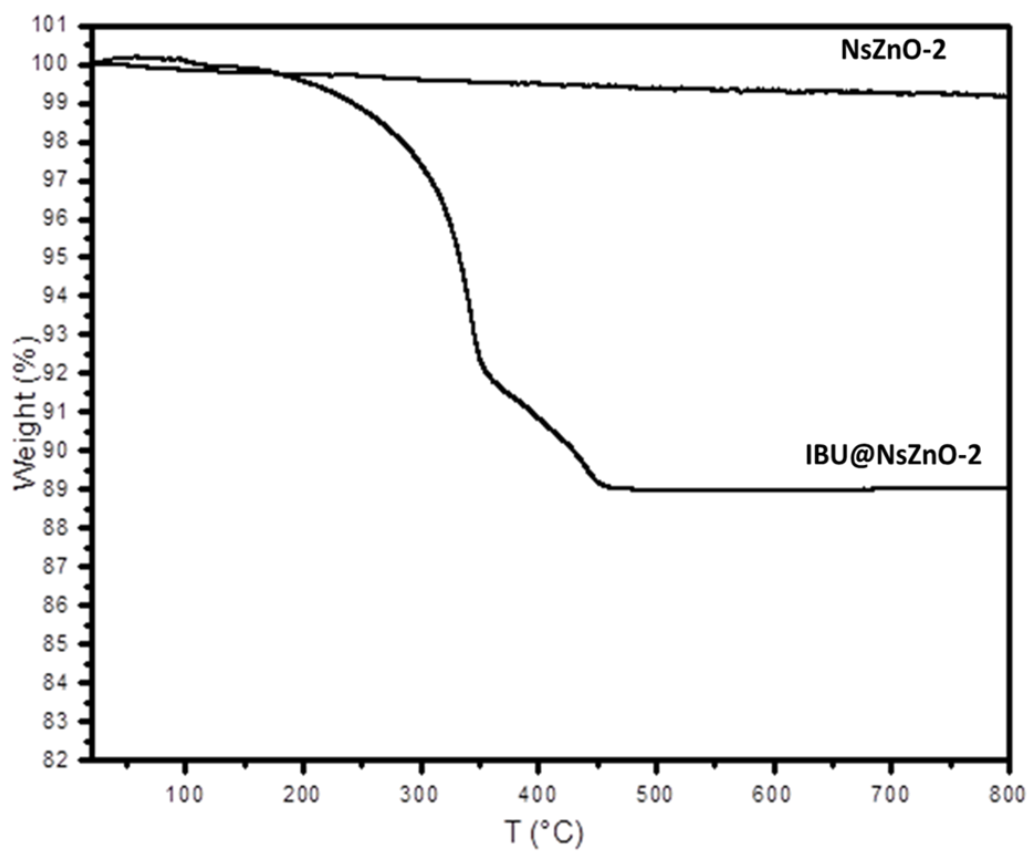

Figure S1 Section A: TG curves of NsZnO-1 and IBU@NsZnO-1; Section B: TG curves of NsZnO-2 and IBU@NsZnO-2.

Supplement: Supplementary file 1 [file nanomaterials-09-00407-s001.pdf]
